# iTBS Stimulation of the Bilateral IFG/IPL Alters the Oscillatory Pattern in ASD

**DOI:** 10.3390/brainsci16020192

**Published:** 2026-02-06

**Authors:** Mitra Assadi, Reza Koiler, Ryan Ally, Richard Fischer, Rodney Scott

**Affiliations:** 1 Neuroscience Department, Thomas Jefferson University, 901 Walnut St., Suite 400, Philadelphia, PA 19107, USA; 2 Neuromechanix, Newark, DE 19713, USA; rkoiler@neuromechanix.com; 3 Department of Psychiatry, Christiana Care Hospital, Wilmington, DE 19718, USA; ryan.ally@christianacare.org; 4 Neuroscience Department, Christiana Care Hospital, Wilmington, DE 19718, USA; rfischer@christianacare.org; 5 Neurology and Pediatrics, Thomas Jefferson University, Philadelphia, PA 19107, USA; rodney.scott@nemours.org; 6 Pediatric Neurology, AI Dupont Children’s Hospital, Wilmington, DE 19803, USA

**Keywords:** ASD, iTBS, mirror neurons, oscillatory pattern

## Abstract

Background: Autism Spectrum Disorder (ASD) is a neurodevelopmental condition characterized by impairments in social communication, reciprocity, and adaptive behavior. Converging neurobiological evidence suggests that these clinical features arise from aberrant connectivity and dysregulated neuronal oscillations across distributed brain networks. In particular, dysfunction within the mirror neuron regions, concentrated in the inferior frontal gyrus (IFG) and inferior parietal lobule (IPL), has been implicated in deficits of imitation, empathy, and social cognition in ASD. Non-invasive neuromodulation using repetitive transcranial magnetic stimulation (rTMS) has shown modest behavioral benefits in ASD. However, most studies apply the conventional protocols targeting the dorsolateral prefrontal cortex. The effects of intermittent theta-burst stimulation (iTBS), a potent excitatory rTMS protocol targeting the mirror neuron regions, on the oscillatory dynamics in ASD remain largely unexplored. Objective: To investigate whether iTBS targeting the bilateral IFG and IPL modulates EEG-derived oscillatory activity in adolescents with ASD and to explore the relationship between oscillatory changes and social reciprocity. Methods: Six adolescents with Level I or II ASD (ages 13–18) underwent bilateral iTBS targeting the IFG and IPL using a figure-of-eight coil and standardized theta-burst parameters. Participants were randomized to receive either 18 active iTBS sessions or a waitlist-controlled crossover design (9 sham followed by 9 active sessions). Standard 21-channel EEG recordings were obtained during the first (EEG-1) and final (EEG-2) active stimulation sessions, including pre- and post-stimulation epochs. Power spectral analyses were conducted across frequency bands (delta through gamma). Behavioral outcomes were assessed using the Childhood Autism Rating Scale, Second Edition (CARS2), administered pre- and post-intervention. Results: All participants tolerated the intervention without adverse effects. Behavioral analysis demonstrated a significant reduction in CARS2 scores following iTBS and is reported in detail in our prior clinical outcomes manuscript, consistent with improved social reciprocity (*p* < 0.001). EEG analysis revealed an immediate post-stimulation increase in gamma-band power during EEG-1 in five of six participants, whereas lower-frequency bands exhibited variable responses. In contrast, EEG-2 showed no consistent post-stimulation gamma enhancement. Net comparisons between EEG-1 and EEG-2 demonstrated attenuation of the initial gamma response in the same five participants. At the group level, gamma percent change did not reach statistical significance at EEG-1 (*p* = 0.12) or EEG-2 (*p* = 0.66), and exploratory comparisons between the 9-active versus 18-active arms did not reach statistical significance. While ipsi-directional changes in gamma power and CARS2 scores were observed in four participants, correlation was not identified in this pilot sample. Conclusions: Bilateral iTBS targeting the IFG and IPL induces a transient enhancement of gamma oscillations in adolescents with ASD that attenuates with repeated stimulation. This pattern is consistent with adaptive homeostatic plasticity (metaplasticity) within excitatory–inhibitory circuits, potentially mediated by GABAergic interneurons. These findings support the feasibility of EEG as an objective biomarker of neuromodulatory engagement in ASD and highlight the importance of network-level and oscillatory mechanisms in interpreting therapeutic responses. Larger, sham-controlled studies incorporating multimodal biomarkers are warranted to clarify clinical relevance and optimize personalized neuromodulation strategies.

## 1. Introduction

Autism Spectrum Disorder (ASD) encompasses a wide range of limitations in reciprocal, social, and communicative milestones, as well as restrictive/repetitive behaviors, leading to significant life-time challenges. ASD is a prevalent neurodevelopmental condition with staggering clinical, social, and financial burdens. Contemporary epidemiological data indicate a rising prevalence, underscoring the growing clinical, societal, and economic burden associated with ASD [1]. Importantly, ASD is not a unitary disorder but rather a highly heterogeneous spectrum, with marked interindividual variability in symptom severity, cognitive profile, adaptive functioning, and developmental trajectory. This heterogeneity strongly suggests that ASD arises from complex and interacting neurobiological mechanisms [2].

Early neurobiological models of ASD emphasized localized cortical dysfunction. However, advances in neuroimaging, neurophysiology, and connectomics have shifted the field toward a network-based model. Within this framework, ASD is increasingly conceptualized as a disorder of distributed brain networks characterized by atypical connectivity, altered synaptic plasticity, and dysregulated neuronal oscillations [3].

Among the neural networks implicated in ASD, the mirror neuron system has received sustained attention due to its putative role in social cognition, imitation, empathy, and action understanding [4,5,6,7,8,9]. Core components of this system are localized primarily within the inferior frontal gyrus (IFG) and inferior parietal lobule (IPL) [5], regions that are structurally and functionally interconnected via long-range white matter tracts, including the superior longitudinal fasciculus, forming a core substrate for the mirror neuron system and the broader social reciprocity circuitry [10]. Structural neuroimaging studies have demonstrated reduced gyrification and atypical cortical maturation in the IFG extending into the IPL in individuals with ASD [11]. Functional MRI and neurophysiological investigations further reveal reduced coherence, abnormal synchronization, and altered functional connectivity within IFG–IPL circuits, with these abnormalities correlating with deficits in imitation, empathy, and reciprocal social interaction [12].

Beyond mirror neuron network abnormalities, ASD is associated with widespread microstructural and macrostructural brain alterations, including early brain overgrowth, atypical synaptic pruning, and disrupted long-range connectivity [13,14,15,16]. These changes result in inefficient neuronal circuitry, which compromises the oscillatory coordination required for high-level social cognition. Oscillatory activity across canonical frequency bands (delta, theta, alpha, beta, and gamma) supports hierarchical organization of brain function. A converging body of evidence implicates dysregulated neuronal oscillations as a unifying mechanism through which such circuit-level abnormalities manifest at the systems level [17,18].

Despite extensive behavioral and neurobiological characterization of ASD, pharmacological treatments targeting core symptoms remain limited in efficacy. This therapeutic gap has driven increasing interest in noninvasive brain stimulation techniques, particularly repetitive transcranial magnetic stimulation (rTMS) [19]. The rTMS is a promising tool in ASD [20] with well-established safety in the pediatric population [21,22]. The meta-analyses suggest a modest improvement in some behavioral measures post-rTMS in ASD [23,24]. While the protocols in these studies varied considerably, many used low-frequency stimulation on the dorsolateral prefrontal cortex (DLPFC), largely extrapolating from protocols developed for mood and executive dysfunction. In contrast, relatively few studies have targeted IPL or IFG in ASD despite their central role in social cognition [25,26,27,28,29]. Given the crucial involvement of these regions within the mirror neuron system and broader social brain networks, neuromodulation of the IFG/IPL may yield mechanistically distinct and potentially more relevant effects on social cognition compared with DLPFC stimulation.

A recent systematic review of the literature on the application of rTMS in ASD published in 2024 revealed that while the majority of the researchers continue to target the DLPFC, IPL is emerging as a new target. The review concluded that the intervention resulted in discernible enhancement across a spectrum of scales [30].

Application of rTMS may include a variety of excitatory or inhibitory protocols; intermittent theta burst stimulation (iTBS), a patterned rTMS protocol, produces a robust excitatory effect, and induces durable synaptic plasticity via mechanisms analogous to long term potentiation [31]. Despite its potency, iTBS remains underutilized in ASD, accounting for only 20% of the research in this field [30].

Current ASD interventions rely on behavioral scales, which, while clinically meaningful, offer limited insights into the underlying pathophysiology. Neurophysiological biomarkers to capture the impact of neuromodulation at the network level are critically needed in ASD. Electroencephalography (EEG) based oscillatory and connectivity measures provide objective and quantifiable biomarkers of the neuronal circuitry. EEG measures neural oscillations across delta (1–4 Hz), theta (4–8 Hz), alpha (8–13 Hz), beta (13–30 Hz), and gamma (>30 Hz) bands. Previous studies have demonstrated alterations across a wide range of spectral frequencies at baseline in ASD. An increase in the gamma power at rest and an overall dysregulation of gamma oscillation have been reported in ASD [32,33].

Despite the above knowledge, the impact of iTBS on oscillatory patterns remains understudied in ASD. This pilot study addresses this gap by examining the effects of iTBS targeting the bilateral IFG and IPL on EEG-derived oscillatory activity in adolescents with ASD. We aimed to test the hypothesis that iTBS application to the bilateral mirror neuron regions (IFG and IPL) will produce a modulatory effect on the oscillatory patterns as measured on the EEG and will be accompanied by behavioral improvements.

## 2. Methods

### 2.1. Study Design and Participants

This pilot study was funded by the Delaware Health Science Alliance (NCT06807684, https://clinicaltrials.gov/study/NCT06807684) and conducted at Christiana Care Health System Neuroscience Department in Delaware. The protocol was approved by the Institutional Review Board, and informed consent was obtained from all participants and their parents. Adolescents with ASD (Level I or II; ages 13–18) were recruited. The inclusion/exclusion criteria are shown in Table 1.

### 2.2. Randomization and Experimental Design

Participants were randomized to receive either 18 active iTBS sessions or a waitlist-controlled crossover design (9 sham followed by 9 active sessions). Stimulation was delivered bilaterally to IFG and IPL using a figure-of-eight coil Magstim^®^ (Whitland, UK) Horizon^®^ (Heath, TX, USA) equipment, utilizing 55% of the maximum stimulator output for all patients [34]. The participants received the iTBS standard protocol (quick bursts of 3 pulses at 50 Hertz for a total of 600 pulses per target delivered over 4 min), 2400 pulses per session total, divided equally between the bilateral IFG and IPL. Target localization was guided by a commercial EEG cap designed based on the standard 10–20 electrode placement system, with IFG approximated to the F5/6 and IPL to the P3/4 electrodes [35,36]. This approach is a widely used method for targeting in the absence of neuro-navigation, particularly in pediatric ASD, where tolerability is a key consideration. Details regarding the methodology and behavioral outcomes were recently published in a separate manuscript [37].

### 2.3. Outcome Measures

Childhood Autism Rating Scale, 2nd edition, high functioning (CARS2): A 15-item scale for ASD Level I or II [38], was administered by a neuropsychologist pre- and post-intervention.

EEG: Standard 21-channel EEG studies were recorded during the first (EEG-1) and last (EEG-2) active sessions and included 5 min pre- and post-stimulation epochs. We utilized the standard 10–20 electrode placement system, implementing the following parameters: impedances < 5 kilo ohm, sensitivity of 7 microvolts/mm, 1 Hertz high- pass and 70 Hertz low- pass filters with a sampling rate of 256 Hertz. For participants in the Waitlist/Active arm, EEG-1 corresponded to session 10 (the first active session after the 9 sham sessions), and EEG-2 corresponded to session 18 (the ninth active session). No EEG was acquired at the end of the sham block; therefore, any sham carryover effect on the first active-session EEG cannot be isolated in the present dataset.

### 2.4. Data Analysis

The results of the CARS2 assessments were compared before and after the intervention, as published in our recent manuscript [37].

The EEGs were visualized using Matlab’s (manufactured by MathWorks, Natick, MA, USA) basic plotting functions. Power spectra for each epoch were estimated by dividing the period into 2 s intervals and calculating the power spectra of each interval using Matlab’s Fast Fourier Transform (version R2024b), analyzing neural oscillations across delta (1–4 Hz), theta (4–8 Hz), alpha (8–13 Hz), beta (13–30 Hz), and gamma (>30 Hz) bands. Power, defined as one half of the square amplitude, was calculated for the 2 s intervals and averaged.

Raw power spectra demonstrated spikes at certain frequencies of known artifactual origin, such as the 60 Hz commercial electric line noise and the 75 Hz refresh rate of computer screens. Therefore, a thresholding method based on the mean and standard deviation of the power spectra above the appropriate frequency was used to remove such spikes. We selected the power spectra greater than or equal to 1 Hz. The sampling frequency of 256 Hz was used to determine a maximum frequency of 128 Hz (the Nyquist frequency). The time interval of 2 s conferred a resolution of 0.5 Hz in the estimated power spectra.

Changes in spectral power were calculated as percentage differences between pre- and post-stimulation epochs for each channel and used to compute the average for all channels. Net differences were determined by subtracting EEG-1 averages from EEG-2. Statistical analyses were performed on the subject-level, all-channel average percentage changes. For each timepoint (EEG-1 and EEG-2), one-sample *t*-tests evaluated whether the mean gamma percent change differed from zero. A paired *t*-test (equivalently, a one-sample *t*-test on EEG-2 minus EEG-1) evaluated attenuation across sessions. To assess potential confounding by study arm (Active/Active vs. Waitlist/Active), exploratory arm-stratified comparisons were performed using Welch’s *t*-tests and exact permutation tests (all 20 possible 3-versus-3 allocations). Pearson and Spearman correlations were computed between the attenuation metric (EEG-2 minus EEG-1 gamma percent change) and CARS2 change. All tests were two-tailed with α = 0.05 and are reported as exploratory given the pilot sample size.

## 3. Results

Six adolescents with ASD (mean age of 14.8 and standard deviation of 1.9 years), 3 female and 3 male, were enrolled between October 2024 and June 2025 (Table 2). No adverse effects were noted. CARS2 scores declined significantly post-intervention, consistent with improved social reciprocity (*p* < 0.001) [37].

**Figure 1:**
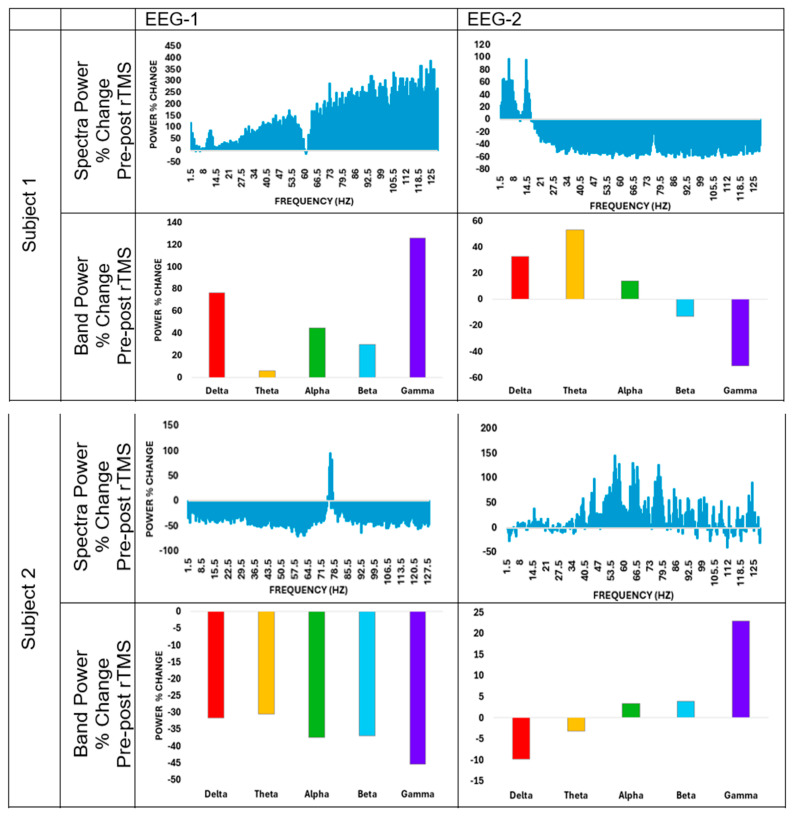

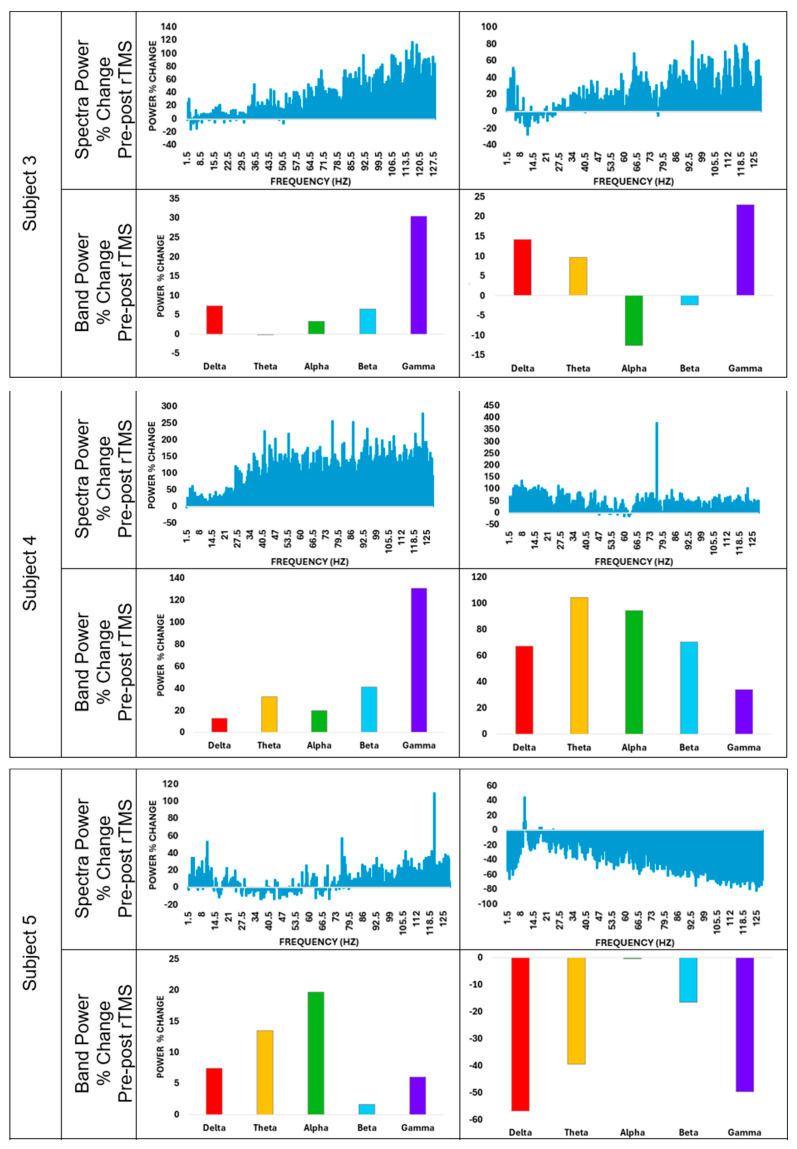

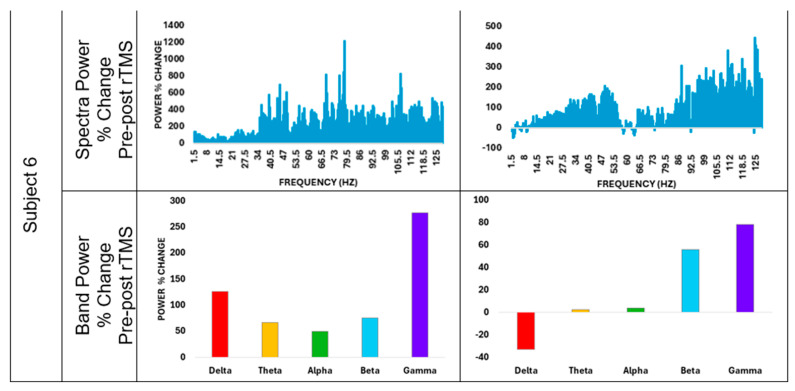
The average spectra and band power percentage changes for all channels on EEG-1 and EEG-2.

Figure 1 illustrates the average power spectra and percentage changes across canonical frequency bands for all EEG channels during EEG-1 and EEG-2 sessions. During EEG-1, five of six participants demonstrated an immediate post-stimulation increase in gamma-band power, while lower-frequency bands (delta through beta) showed heterogeneous responses. This pattern suggests a preferential engagement of fast oscillatory dynamics following initial exposure to iTBS. In contrast, EEG-2 did not reveal a reproducible post-stimulation gamma enhancement, indicating a marked reduction in the immediate oscillatory responsiveness following repeated stimulation. Across subjects, gamma percent change at EEG-1 ranged from −45.4% to +277.2%, whereas gamma percent change at EEG-2 ranged from −51.1% to +78.1%. Delta through beta band responses varied in both magnitude and direction across subjects at both timepoints.

**Figure 2:**
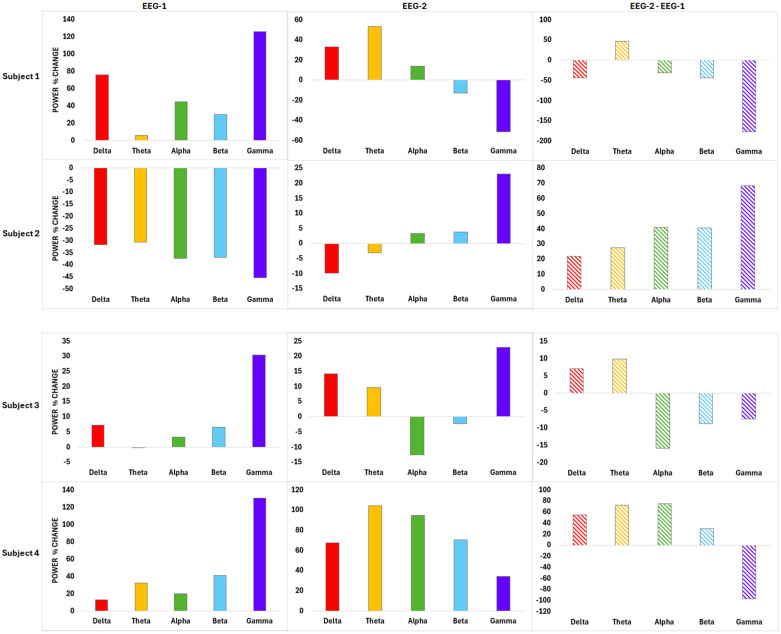

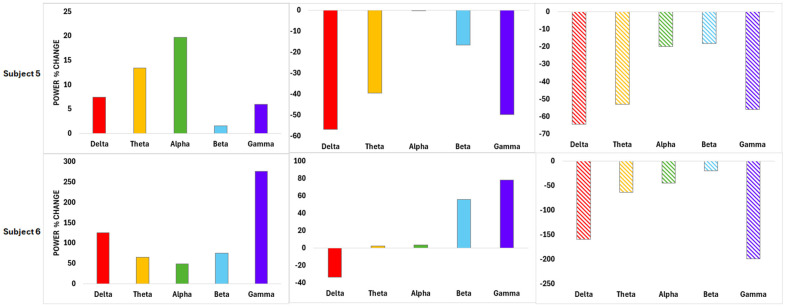
Power spectra from left to right: delta, theta, alpha, beta, and gamma. Columns correspond to EEG-1, EEG-2, and the net change. The net change is shown with stripes, gamma is shown in purple. Net change in post–pre band power shows percent change between sessions (EEG-2 minus EEG-1) for each subject. Negative values indicate attenuation of the immediate post-stimulation response at the final active session relative to the first active session. Gamma-band net changes (EEG-2 minus EEG-1) were: Subject 1, −177.0%; Subject 2, +68.4%; Subject 3, −7.5%; Subject 4, −96.5%; Subject 5, −55.8%; Subject 6, −199.1%.

Figure 2 presents the net change in gamma-band power by comparing post–pre differences between EEG-1 and EEG-2. Five participants exhibited clear attenuation of gamma enhancement during EEG-2 relative to EEG-1, represented by negative net changes, suggesting a dose-dependent adaptive response to repeated iTBS. One participant demonstrated a divergent response, highlighting interindividual heterogeneity that may reflect baseline neurophysiological differences.

A one-sample *t*-test was carried out to assess whether the mean gamma-band percent change (post–pre) differed from zero at each timepoint. The group-level mean gamma change did not reach significance at EEG-1 (mean 87.5%, 95% CI [−33.9, 208.8], *p* = 0.12) or EEG-2 (mean 9.5%, 95% CI [−43.7, 62.8], *p* = 0.66). The within-subject attenuation metric (EEG-2 minus EEG-1) had a negative mean (mean −77.9%, 95% CI [−184.6, 28.8]) but did not reach significance (*p* = 0.12). Exploratory arm-stratified comparisons (Active/Active vs. Waitlist/Active) did not reach statistical significance for EEG-1, EEG-2, or the attenuation metric (Welch’s *p* = 0.26, 0.08, and 0.64, respectively; exact permutation *p* = 0.20, 0.20, and 0.70, respectively). While the attenuation metric and CARS2 change were ipsi-directional in 4/6 subjects, correlation was not identified (Pearson r = −0.04, *p* = 0.94; Spearman ρ = 0.03, *p* = 0.95).

## 4. Discussion

This exploratory pilot study demonstrates that iTBS targeting the bilateral IFG/IPL may be associated with transient modulation of the gamma band oscillatory activity in adolescents with ASD. Specifically, an immediate enhancement of gamma power was observed following initial stimulation, which attenuated after repeated sessions. These neurophysiological changes were accompanied by improvements in social reciprocity as measured by CARS2, although direct correlations were limited by sample size.

EEG reflects the summation of excitatory and inhibitory postsynaptic potentials in the cortical neurons. The rhythmic nature of neural activity, manifested in different frequency oscillations, is governed by the harmonic synchronization of the postsynaptic potentials in various neuronal populations. Neurons participating in these synchronized assemblies demonstrate temporally aligned oscillations, which, at the circuit level, orchestrate network organization and response modulation. Previous studies have demonstrated alterations across a wide range of spectral frequencies at baseline in ASD. While some authors have reported an increase in the gamma power at rest and a decline during cognitive processing tasks in ASD [32] others have proposed reduced resting-state gamma power in ASD compared to neurotypical individuals [39].

iTBS-induced modulation is measurable via quantifying motor evoked potential (MEP) amplitudes. iTBS can induce MEP amplitude facilitation by 35% for up to an hour. The recovery of the amplitude of the MEP to baseline is proposed as an index for neuronal plasticity induced by iTBS [31,40,41]. Compared to the neurotypical individuals, ASD patients exhibit greater and longer-lasting effects after iTBS [40,42,43,44].

We propose that our observations may represent an adaptive response to iTBS, attributable to neuronal plasticity. Neuronal plasticity, characterized by the brain’s dynamic capacity to remodel the networks in response to neuronal activity, has a critical role in the maturation of the nervous system, in experience-dependent learning, and adaptation to injuries. Excitatory glutamatergic signaling, as well as the inhibitory activity of the fast-spiking GABAergic inhibitory interneurons, are crucial for orchestrating neuronal plasticity [45]. rTMS influences neuronal plasticity by modulating glutamatergic and GABAergic pathways [46,47]. In particular, iTBS mimics neural oscillations associated with Hebbian plasticity and is known to produce an excitatory modulatory effect and long-term potentiation [46,48,49,50].

While the underlying pathophysiology in ASD is heterogeneous, an imbalance between the excitatory and inhibitory signaling (E/I imbalance) has been introduced as a unifying framework allowing convergence of various abnormalities into a common final pathway [18]. In vivo analyses of the neurotransmitter metabolites using proton spectroscopy have consistently demonstrated decreased GABA in the peri-Rolandic and temporal regions in ASD. However, the measurements of glutamate/glutamine levels have not yielded concrete results. Magnetic resonance spectroscopy studies have shown a direct correlation between reduced GABA levels and the clinical features of ASD [47,51,52]. The recent use of functional MR spectroscopy in exploring GABA/glutamate dynamics while processing social tasks may enhance our understanding of E/I imbalance in ASD [53]. These findings have led to a concept of inhibitory deficit, in part attributed to the paucity of the fast-spiking GABAergic inhibitory interneurons in ASD [18]. In this context, the initial gamma enhancement in our study may reflect heightened network engagement in a system characterized by baseline hyperexcitability.

The gamma power has been proposed as a proxy for the E/I balance, as it is directly modulated by GABA signaling [18,51]. Loss of the inhibitory GABAergic activity results in desynchronization of the gamma oscillations. Desynchronized gamma leads to overactivity of multiple networks with low precision and precludes efficient processing of the salient stimuli, including complex social cues.

Due to the geometrical horizontal orientation of the fast-spiking GABAergic inhibitory interneurons in the cortex, these cells may be more susceptible to neurostimulation [33]. As such, the excitatory effect of iTBS on these interneurons may enhance the GABAergic output and engage the neuroplasticity cascade, ultimately leading to changes in the functional architecture of the neuronal networks, as suggested by our results.

The observations produced by our small pilot project suggest that iTBS modifies the brain-wide oscillatory behavior in neuronal networks in an exposure-dependent fashion. While the naive brain, when perturbed by the first application of iTBS, demonstrated an increase in gamma power, after multiple sessions of the intervention, this impact was dampened. We propose that the attenuation of the gamma response may represent homeostatic plasticity, a regulatory mechanism by which neural circuits dynamically adjust their plastic potential to preserve network stability. Metaplasticity governs the threshold for future synaptic modification based on prior activity, thereby preventing excessive excitation or depression within cortical networks. Importantly, metaplasticity has been demonstrated to operate prominently within inhibitory circuits, particularly the fast-spiking GABAergic interneurons that regulate gamma oscillations [54]. In the context of iTBS, an intervention known to robustly facilitate excitatory synaptic efficacy [46] such compensatory downscaling is thought to reflect adaptive recalibration rather than loss of responsiveness. Given the well-described excitation–inhibition imbalance in ASD, repeated iTBS may initially amplify gamma synchrony in a hyperexcitable system, followed by recruitment of inhibitory homeostatic mechanisms that dampen subsequent responses.

The clinical meaningfulness of our findings remains elusive. The CARS2 scores improved significantly in 5 out of 6 cases, and ipsi-directional changes with the gamma power were noted in 4 out of 6 cases, highlighting the need for additional studies to clarify the clinical relevance of these findings. Interestingly enough, the behavioral improvements occurred despite attenuation of gamma responses, suggesting that sustained excitation is not required for clinical benefit. Importantly, EEG1 captured an acute within-session percent change (post–pre iTBS), whereas the behavioral outcome reflects cumulative change across the intervention. A transient neurophysiological response can plausibly initiate longer-lasting synaptic and network adaptations, such as changes in baseline excitability, connectivity, or cross-frequency coupling. In this framework, attenuation of the acute gamma response over repeated sessions may reflect homeostatic recalibration (metaplasticity) rather than loss of therapeutic effect. Accordingly, acute gamma modulation may be better interpreted as a biomarker of target engagement and network perturbation rather than a direct surrogate for durable clinical improvement. In brief, the transient perturbation followed by adaptive network reorganization may represent a more relevant mechanism for the therapeutic change, and the gamma modulation may serve as a biomarker of neuronal engagement rather than a direct surrogate of the clinical outcome.

EEG-TMS paradigms have moved from fixed open-loop to adaptive closed-loop systems, using real-time EEG feedback to dynamically adjust rTMS parameters. For instance, a novel TMS-EEG paradigm applying iTBS to the right posterior superior temporal sulcus has been used to develop a putative ASD marker involving face recognition [55]. These approaches enable personalized, state-dependent brain modulation, especially with the help of artificial intelligence and machine learning, to optimize the protocols [56], aiming for precision and enhanced efficacy compared to the conventional methods [57,58].

## 5. Study Limitations and Future Directions

We acknowledge that the very small sample size limits the generalizability of our findings. By the same token, we were unable to reach statistical significance. While our study was based on a waitlist-controlled crossover design, it lacked a fully sham-controlled group. EEG was not acquired immediately before and after the sham block in the Waitlist/Active arm; therefore, sham carryover (if any) on acute gamma responsiveness at the first active session cannot be separated from baseline heterogeneity or nonspecific time/visit effects. Our study was also limited by the short duration of the experiment.

Additional studies recruiting a larger randomized cohort and longer follow-ups are necessary to confirm the neuro-modulatory effects of iTBS stimulation on the mirror neuron regions in ASD. Further confirmation and exploration of this topic may be facilitated by multi-modal biomarkers such as MR spectroscopy to assess neurotransmitter dynamics.

With continuous investigation of neurostimulation and the biological underpinnings of ASD, as well as further development of closed-loop intervention devices, we may be able to realize the progress needed for highly individualized treatment approaches for ASD patients.

## 6. Conclusions

While this exploratory pilot study did not reach statistical significance, the results suggest that iTBS targeting the mirror neuron regions in ASD may produce a transient enhancement of gamma oscillations, which attenuates with repeated exposure, consistent with adaptive neuroplastic mechanisms. These neurophysiological changes were accompanied by an improvement in social reciprocity, elucidating the feasibility of EEG- informed neuromodulation as a promising tool for tailoring individualized ASD interventions.

## Figures and Tables

**Table 1:** Inclusion and Exclusion Criteria.

**Inclusion Criteria:**
1. Meeting the diagnosis of ASD level I or II as confirmed by the CARS2, HF
**Exclusion Criteria:**
1. Patients with ASD exhibiting significant anxiety or contact avoidance, precluding them from cooperating with the procedure
2. Patients with a known diagnosis of seizures
3. Presence of any metallic implants or devices in the head or neck area
4. Pregnant women

**Table 2:** Study Subjects.

Subjects Age/Gender	Diagnosis	Randomization	Number of iTBS Sessions	CARS2 Score Change
17/F	ASD II	Active/Active	18	−1.5
14/M	ASD I	Active/Active	18	−1.5
13/M	ASD I	Waitlist/Active	9	−1
13/M	ASD II	Waitlist/Active	9	+1
14/F	ASD I	Active/Active	18	−2
18/F	ASD II	Waitlist/Active	9	−1.5

CARS2: Childhood Autism Rating Scale, 2nd edition. A decrease in the scores consistent with improvement in social reciprocity was noted in 5/6 subjects; statistical analysis of clinical outcomes is reported in detail in our prior manuscript (*p* < 0.001).

## Data Availability

The original data presented in this manuscript are openly available on Zenodo, https://doi.org/10.5281/zenodo.16970320. iTBS stimulation of the bilateral IFG/IPL alters the oscillatory pattern in ASD.
